# Generation of Cell Lines to Complement Adenovirus Vectors using Recombination-Mediated Cassette Exchange

**DOI:** 10.1186/1472-6750-10-92

**Published:** 2010-12-23

**Authors:** Susan J Morris, Daniel C Farley, Keith N Leppard

**Affiliations:** 1 Department of Biological Sciences, University of Warwick, Coventry, CV4 7AL, UK; 2 Jenner Institute, ORCRB, University of Oxford, Off Roosevelt Drive, Headington, Oxford, OX3 7DQ, UK; 3 Oxford Biomedica (UK) Ltd., Medawar Centre, Oxford Science Park, Oxford, OX4 4GA, UK

## Abstract

**Background:**

Adenovirus serotype 5 (Ad5) has many favourable characteristics for development as a gene therapy vector. However, the utility of current Ad5 vectors is limited by transient transgene expression, toxicity and immunogenicity. The most promising form of vector is the high capacity type, which is deleted for all viral genes. However, these vectors can only be produced to relatively low titres and with the aid of helper virus. Therefore a continuing challenge is the generation of more effective Ad5 vectors that can still be grown to high titres. Our approach is to generate complementing cell lines to support the growth of Ad5 vectors with novel late gene deficiencies.

**Results:**

We have used LoxP/Cre recombination mediated cassette exchange (RMCE) to generate cell lines expressing Ad5 proteins encoded by the L4 region of the genome, the products of which play a pivotal role in the expression of Ad5 structural proteins. A panel of LoxP parent 293 cell lines was generated, each containing a GFP expression cassette under the control of a tetracycline-regulated promoter inserted at a random genome location; the cassette also contained a LoxP site between the promoter and GFP sequence. Clones displayed a variety of patterns of regulation, stability and level of GFP expression. Clone A1 was identified as a suitable parent for creation of inducible cell lines because of the tight inducibility and stability of its GFP expression. Using LoxP-targeted, Cre recombinase-mediated insertion of an L4 cassette to displace GFP from the regulated promoter in this parent clone, cell line A1-L4 was generated. This cell line expressed L4 100K, 22K and 33K proteins at levels sufficient to complement L4-33K mutant and L4-deleted viruses.

**Conclusions:**

RMCE provides a method for rapid generation of Ad5 complementing cell lines from a pre-selected parental cell line, chosen for its desirable transgene expression characteristics. Parent cell lines can be selected for high or low gene expression, and for tight regulation, allowing viral protein expression to mirror that found during infection. Cell lines derived from a single parent will allow the growth of different vectors to be assessed without the complication of varying complementing protein expression.

## Background

Currently, 24% of gene therapy clinical trials worldwide are using adenovirus serotype 5 (Ad5) as the delivery vehicle [1]. First generation Ad5 vectors were created by deletion of the genes for the viral transactivator (E1A) and E1B proteins to render the vector replication-incompetent and deletion of the E3 genes, the products of which are non-essential for *in vitro *growth [2]. These vectors have capacity for up to ~7 kbp transgene sequence, can grow to high titres in E1-complementing cells and have the ability to infect a wide range of cells. However, although the ability of these vectors to replicate is significantly inhibited compared to wt virus, they still exhibit low levels of viral late protein expression, and transgene expression *in vivo *is only transient [2]. This is in part because the vector DNA does not integrate and therefore has no mechanism of maintenance in a dividing cell population. However, transience is primarily due to the elimination of cells by the initiation of an immune response to viral vector gene expression products [3]. Second generation vectors are rendered more completely replication-incompetent by the further mutation of E2 or E4 genes [4-6]. Whilst these give improved persistence of transgene *in vivo *because of reduced immune responses, the stability and level of transgene expression is variable [7-11]. The most promising Ad5 vectors for long-term gene delivery *in vivo *have been those that do not contain any viral coding sequence [12,13]. However, to grow these gutted vectors, they must be complemented with helper viruses as no cell line expressing the full array of viral proteins is available; although yields of vector from such systems can be high, there is the additional problem that the helper virus must be inactivated or removed before the vector can be used and residual contamination with helper is likely [14,15]. Therefore, a vector which retained the practical advantages of earlier Ad5 vectors but displayed further reduced viral gene expression and hence induced less toxicity and reduced inflammatory and immune responses would be an ideal gene delivery vehicle. For reviews on adenovirus vectors see [14,16].

The Ad5 L4 region encodes three non-structural proteins, L4-100K, -22K and -33K, which have been shown to be essential for structural protein expression in the late phase of infection. L4-22K acts both at the level of transcription and post-transcriptionally to support late mRNA production [17]. In addition, this L4 protein has been shown to be a packaging factor [18]. L4-33K is a splice factor that is essential for production of a subset of late mRNAs [19,20]. L4-100K is responsible for selective translation of late mRNAs [21] and for the stabilisation and assembly of hexon trimers, which form the major part of the capsid of progeny particles [22]. As might therefore be expected, an L4 100K-mutated, E1^-^, E3^- ^vector was previously reported to have reduced liver toxicity in mice [23]. A vector deleted for all three L4 proteins would still retain the ability to replicate its DNA, and hence to express transgenes to high level, but would be further improved by being unable to produce any of the late structural proteins associated with toxicity and the induction of an inflammatory response.

The propagation of new adenovirus vectors is dependent upon the creation of cell lines to provide the proteins necessary for virion production that the vector is unable to encode. The classic method for generating Ad5 vector complementing cell lines is to transfect cells with a construct containing the viral gene under the control of either its own or a heterologous promoter and to select for cells stably expressing the viral protein, either directly or via a co-transfected or linked antibiotic-resistance gene. This method relies on the integration of the complete construct in one or a few random positions within the chromosome. The site of integration of such expression cassettes affects expression of the inserted gene as gene expression in mammalian cells is controlled by chromatin structure [24]. Chromatin remodelling within the transgene, imposed by the adjacent chromatin structure, can result in promoter silencing [25]. Therefore, a large number of cell lines must be isolated and characterised to identify one that has the desired expression characteristics, which can be inconvenient and time consuming. Furthermore, many proteins, including in our experience L4-100K, are toxic to cells when over-expressed. To obtain cell lines expressing such proteins requires gene expression to be made tightly inducible, thus adding another trait which needs to be tested in potential cell lines. L4-100K cytotoxicity likely reflects its effects on host cell translation; this toxicity may explain the very low level of 100K expression previously achieved by standard cell line techniques [23].

Recombination-mediated cassette exchange (RMCE) using LoxP/Cre-mediated recombination [26-28] has been used previously for a wide range of applications including: engineering cell lines to express high levels of recombinant proteins [29,30]; generating cell lines to study panels of mutant proteins [31]; and generating transgenic stem cell lines [32]. RMCE relies on the pre-insertion into the chromosome of a cassette that contains a LoxP site placed between the promoter and coding sequence of a marker gene. Cell clones with the desired characteristics of marker gene expression are first isolated and then used as the parent for insertion of the gene of interest at the LoxP site by Cre-mediated recombination; at the same time the marker gene is displaced from the promoter. Each resulting clone should have the same characteristics of expression as the marker gene in the parent cell line, because the site of insertion into host chromatin will be identical. Chromosomal positioning effects and silencing of the transgene are therefore avoided. Although the initial workload to isolate and characterise the parent cell lines is equal to that used in the conventional method, the choice of an appropriate marker gene can speed up the screening process and, once generated, such cell lines can be used to generate multiple complementing cell lines with reliable expression characteristics.

Here we report the use of LoxP/Cre RMCE to generate an inducible Ad5 L4-complementing cell line. First, a panel of GFP LoxP parent cell lines was generated and characterised with respect to the inducibility and stability of GFP expression. One parent cell line, selected for its favourable properties, was then used to generate a cell line that expressed Ad5 L4 proteins and was able to support productive infection of L4-mutant viruses.

## Results

### Generation of LoxP parent cell lines

**Figure 1:**
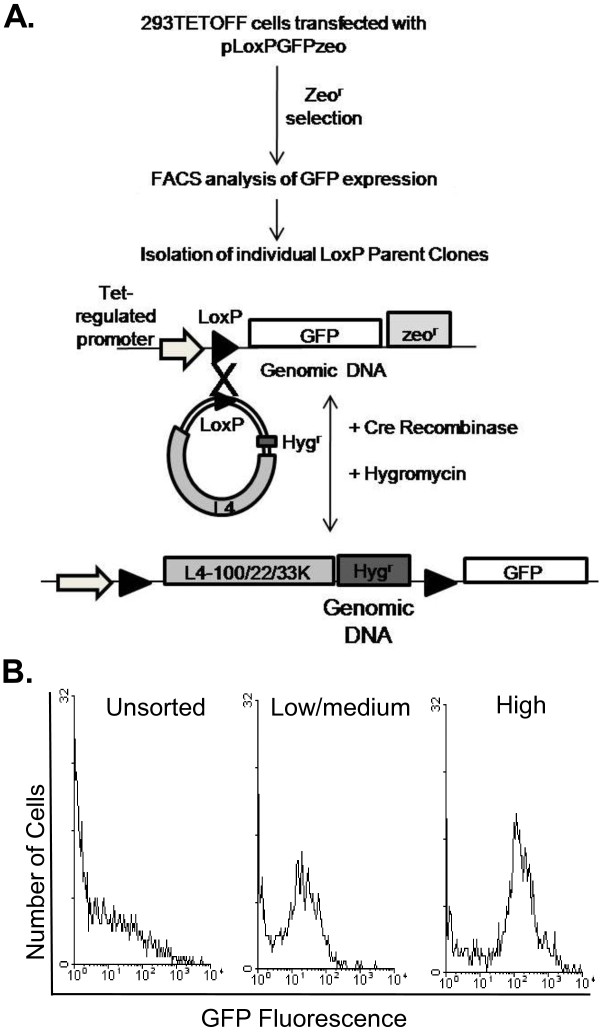
**Select and insert strategy for cell line generation**. (A) Diagram showing the select and insert strategy used to generate L4 complementing cell lines. LoxP parent cell lines are first isolated by selection for Zeocin resistance and characterised for their inducible expression of GFP. Cre-mediated recombination is then used to replace the GFP gene from LoxP parent cells with an L4 cassette from a promoterless shuttle plasmid. (B) To generate LoxP parent cells, 293TETOFF were transfected with pLoxPGFP and Zeocin-resistant cells were sorted by FACS into two populations based on GFP fluorescence intensity. Left: unsorted cell population; centre: cells sorted for low to medium GFP expression; right: cells sorted for high GFP expression.

293 cells are Ad5 E1-complementing cell lines that support the growth of E1^- ^Ad5 vectors to high titres [33]. A derivative of these cells, 293TETOFF, which constitutively expresses the tetracycline transactivator protein tTA, was used as the starting point for the work, with the aim of isolating cell lines exhibiting regulated transgene expression. The overall strategy for creation of Ad5 L4-expressing cell lines from 293TETOFF cells by LoxP/Cre RMCE is shown in Figure 1A. 293TETOFF cells were first transfected with pLoxPGFP, which carries GFP under the control of the tetracycline-regulated P_Bi _promoter, and selected for Zeocin resistance. A population of cells was obtained that had a broad range of GFP expression levels in the absence of the tetracycline analogue, doxycycline (Dox) (Figure 1B, unsorted); 66% of the population showed little or no GFP expression (< 10 fluorescent units, f.u.). Preliminary experiments had indicated that clones expressing low levels of GFP (10-100 f.u.) typically lost transgene expression by 40 days of selection, probably due to chromatin remodelling and promoter silencing since they remained Zeocin-resistant (data not shown). Therefore, to enrich for cells that might subsequently show stable GFP expression and to reduce the number of cells that were not expressing GFP prior to isolating single-cell clones, the Zeocin-resistant population was sorted into low GFP expression (10-100 f.u.) and high GFP expression (100-10,000 f.u.) populations (Figure 1B). The proportion of cells that did not express GFP in these sorted populations was reduced to 30% and 16% respectively. Individual cell clones (LoxP parent lines) were then isolated from the high-expressing population.

### LoxP parent cell lines show varying GFP regulation and expression

**Figure 2:**
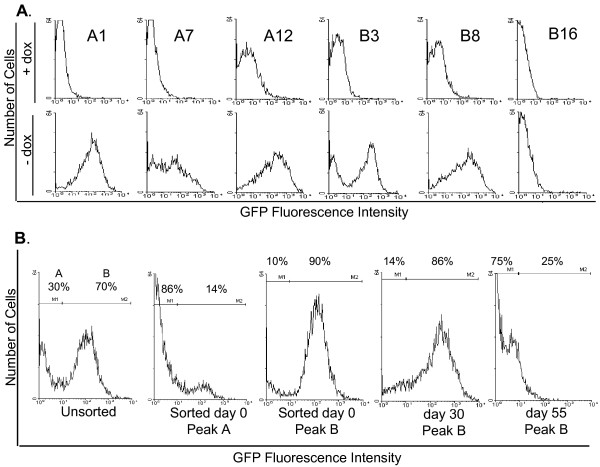
**Analysis of LoxP parent cell lines**. (A) Clones from the high GFP-expressing pool (Figure 1B) were grown in the presence of 100 ng/ml Dox. Media containing Dox was removed 7 days prior to analysis and media changed daily before GFP expression analysis by FACS. (B) Clone B3 contained two sub-populations (A = not expressing GFP; B = expressing GFP), which were separated by FACS. The ability of Clone B3-B cells to be induced to express GFP was analysed at day 0 post-sorting, day 30 and day 55, with Dox removal at each time point as panel A.

The GFP expression characteristics of LoxP parent clones was expected to vary because regulation of the P_Bi _promoter by Dox is dependent upon the site of integration within the chromosome [34]. We wished to obtain a LoxP parent clone that displayed very low basal expression in the presence of Dox, good inducibility upon Dox removal and long-term stability of this inducible expression. 41 LoxP parent cell clones were isolated and tested for their level and regulation of GFP expression; Figure 2A shows a representative selection of these clones. All clones except B16 expressed levels of GFP (induced upon Dox withdrawal) in the range of 100-10,000 f.u., as expected since they were isolated from the high-expressing sorted population (Figure 1B). 21 clones exhibited tight regulation of GFP expression by Dox, e.g. clones A1 and A7 (Figure 2A); tight regulation was defined as less than 5% of cells expressing >10 f.u. GFP (out background cut-off set for non-fluorescent 293TETOFF cells) under conditions of Dox-mediated repression of the GFP transgene. The other 20 clones, in contrast, showed leaky expression in the presence of Dox, e.g. clones A12, B3 and B8 (Figure 2A).

Clone B3 appeared to comprise of two distinct populations, one expressing and one not expressing GFP upon induction (Figure 2A). To determine whether B3 was in fact not clonal or alternatively if its GFP expression phenotype was unstable, the cells were sorted into expressing and non-expressing populations and GFP expression in the expressing population analysed over 55 days. This revealed that B3 GFP expression was unstable, since the proportion of cells induced to express GFP decreased from 90% at day 0 post-sorting to 86% at day 30 and 25% at day 55 (Figure 2B). This instability may be due to heterochromatin assembly on the transgene promoter imposed by the integration context.

### Characterisation of LoxP parent cell line Clone A1

**Figure 3:**
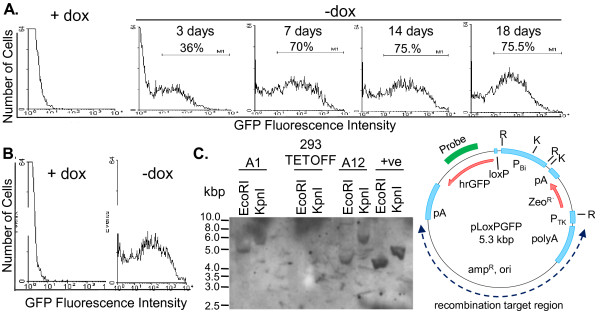
**Characteristics of Clone A1 LoxP parent cell line**. (A) Clone A1 was grown in the absence of Dox for 3, 7, 14 or 18 days with daily media changes before GFP analysis by FACS. (B) Clone A1 was maintained for 60 days in the presence of Dox before removal 7 days prior to analysis of GFP expression. (C) Clone A1, Clone A12, or control 293 TETOFF cell genomic DNA, or pLoxPGFP plasmid DNA (+ve) was digested with EcoRI or KpnI and analysed by Southern blotting using a GFP gene probe. The positions to which DNA size markers migrated are indicated (kbp).

Clone A1 was picked as a potential LoxP parent line from which to generate Ad5 L4-complementing cell lines due to its tight regulation and good inducibility, hence its properties were analysed further. To assess how quickly Clone A1 accumulated GFP after the removal of Dox, the cells were grown in the presence or absence of Dox for 3, 7, 14 or 18 days (Figure 3A). 3 days post Dox removal, 36% of cells expressed GFP, increasing to 70% by day 7 and peaking at 75% by day 14 after the removal of Dox. The low level of recovery of expression by day 3 reflects the difficulty in removing Dox from the cell system, even with daily media washes [35]. Greater than 70% of the cell population expressed GFP for over 11 days showing that transgene expression was stable and prolonged in the absence of Dox. These properties of Clone A1 were stable during 60 days of growth under conditions of Dox repression, with tight promoter regulation in the presence of Dox and a good level of inducible expression of GFP upon Dox withdrawal being maintained throughout (Figure 3B). This level and duration of expression, applied to an Ad5-complementing transgene, should be more than adequate for the growth of adenovirus vectors, which have a 24-36 hour replication cycle in complementing cells and produce plaques within 7-9 days.

To be useful as a parent for cell line generation by a RMCE strategy, it was important that Clone A1 cells contained only one copy of the LoxP-GFP cassette, which would be the target for Cre-mediated insertion of the transgene. To test this, genomic DNA from Clone A1, and Clone A12 included for comparison, was analysed by Southern blot using a probe for the GFP gene (Figure 3C). This probe, which gave specific hybridisation since control cell 293TETOFF DNA gave no bands, detected the expected fragments of 4.1 kbp and 5.1 kbp in EcoRI and KpnI digests of the input plasmid pLoxPGFP. Clone A1 DNA also gave single probe-specific fragments in the two digests, however these were in each case larger than the equivalent fragments from the input plasmid. The increase in size results from the linking of probe target sequence in the plasmid to genomic DNA to create hybrid EcoRI and KpnI fragments. The fact that there was only one such fragment in each digest from the Clone A1 cells indicated that a single integration event had occurred. Clone A12 DNA also gave single major bands in each of the two digests with sizes distinct from those in both Clone A1 and the plasmid control, indicating the presence of a different single integration site in this clone. Two weak additional bands from the A12 EcoRI digest probably arose from incomplete digestion. We did not assess the proportion of isolated clones that carried single insertions of the regulated hrGFP cassette, however the fact that two randomly selected clones had single inserts suggests this frequency was high.

### Generation of A1-L4 cell lines

**Figure 4:**
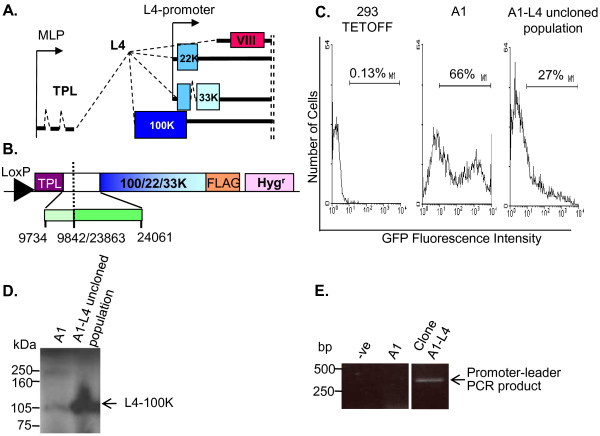
**Generation of A1-L4 cell lines**. (A) The Ad5 major late transcription unit L4 region. During expression from the major late promoter (MLP), leaders 1, 2 and 3 (the tripartite leader; TPL) are spliced to alternative acceptor sites to generate L4 mRNAs encoding 100K, 22K, 33K and the structural protein pVIII; L4-22K and -33K proteins share the same N-terminus but have distinct C-termini due to the presence of an intron in the L4-33K ORF. L4-22K and -33K are also expressed from the L4 promoter early in infection. (B) The L4 cassette within pShuttle100/22/33KFLAG, used for RMCE to generate the A1-L4 cell population. Numbers indicate Ad5 genome positions. LoxP: DNA target sequence for Cre recombinase; FLAG: C-terminal epitope tag on the 33K open reading frame; Hyg^R^; hygromycin-resistance gene. (C) 293TETOFF, Clone A1 and the uncloned A1-L4 population, 21 days post-recombination, were grown without Dox for 3 days with daily media changes and the level of GFP analysed by FACS. M1 shows the percentage of cells expressing GFP above the threshold set by 293TETOFF cell background fluorescence. (D) Clone A1 cells and the uncloned A1-L4 cell population were analysed for L4-100K expression by western blot analysis. The positions to which proteins of known molecular mass migrated are indicated (kDa). (E) To confirm correct insertion of the L4 cassette in clone A1-L4 obtained from the A1-L4 population, genomic DNA from these and control (A1) cells was used as template for PCR amplification using one primer to the tetracycline-regulated P_Bi _promoter and another to the leader 3 sequence within the TPL of the L4 cassette; -ve, PCR negative control. All lanes shown derive from the same exposure of a single gel with irrelevant lanes excised for clarity of presentation. The positions to which DNA size markers migrated are indicated (bp).

The L4 region of Ad5 is part of the major late transcription unit (MLTU) and is expressed from the major late promoter (MLP) during the late phase of infection [36]. Specific mRNAs able to encode L4-100K, -22K or 33K are generated from a single primary transcript by alternative splicing, which links the relevant 3' exons to a tripartite leader sequence (TPL; common to all MLTU mRNAs) comprised of leaders 1, 2 and 3 (Figure 4A). The TPL allows selective translation of mRNA late in infection when host cell shutoff has been initiated [37,38]. To allow creation of inducible L4 cells from LoxP parent Clone A1, a promoterless L4 cassette suitable for Cre-mediated insertion was designed that included the L4-100K, -22K and -33K ORFs. The cassette also included a 5' TPL sequence and, as spliced mRNAs are exported more efficiently from the nucleus than unspliced mRNAs [39], a 306 bp internally truncated intron comprising the TPL leader 3 splice donor and the L4-100K splice acceptor sites (Figure 4B).

Clone A1 cells were transfected with a plasmid containing this cassette (pShuttle100/22/33KFLAG) and pCre to give hygromycin-resistant L4-expressing cells by Cre-mediated recombination. In the uncloned population of A1-L4 cells, analysed 21 days after selection, only 27% of cells expressed GFP 3 days after the removal of Dox compared to 66% of the parental cells (Figure 4C). This loss of GFP expression was accompanied by the gain of L4-100K expression as detected by western blotting (Figure 4D). This pattern of gene expression is consistent with insertion of the L4 cassette at the LoxP site as planned.

To confirm that the L4 cassette had been inserted downstream of the P_Bi _promoter, a single cell clone, Clone A1-L4, was isolated from the A1-L4 population for further characterisation. Genomic DNA from Clone A1-L4 and from its parent A1 was used as template to amplify a PCR product using primers designed against P_Bi _and TPL leader 3 within the L4 cassette. A product will only be obtained if the TPL-L4 cassette is in the correct location so as to bring these two primer binding sites, one from the A1 parent and one from the shuttle plasmid, into proximity. A product of the expected size, 441 bp, was amplified from Clone A1-L4 but not Clone A1 genomic DNA (Figure 4E). Taken together, these data show that recombination has occurred between the genomic DNA and the shuttle plasmid, resulting in the displacement of the GFP gene from the tetracycline-regulated promoter.

**Figure 5:**
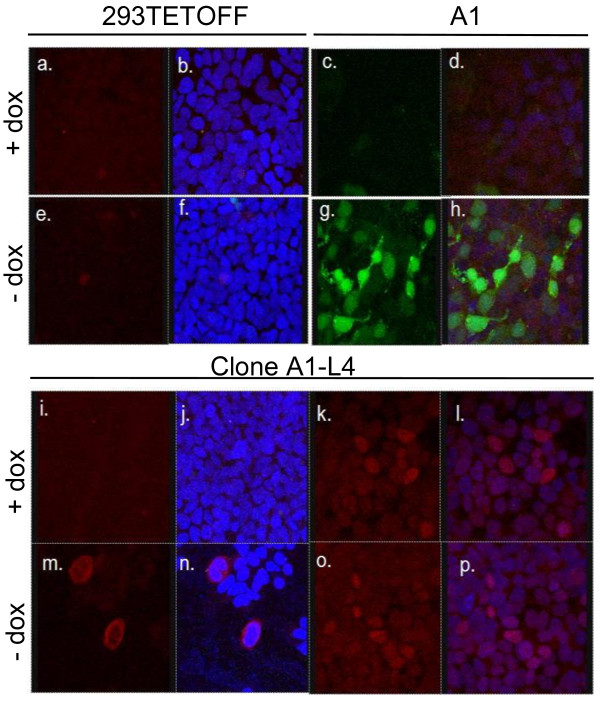
**Analysis of Clone A1-L4 gene expression**. Protein expression from Clone A1-L4 was analysed by fluorescence microscopy. 293TETOFF (a, b, e & f), Clone A1 (c, d, g & h) and Clone A1-L4 cells (i-p) were grown in the presence (a-d & i-l) or absence (e-h & m-p) of Dox for 3 days with daily media changes. Cells were fixed and stained for L4-100K (red, i, j, m & n) or FLAG-tagged L4-33K (red, a-h, k, l, o & p) and nuclear DNA (DAPI, blue) and visualised using a Leica SP2 confocal microscope. GFP autofluorescence was also imaged (green). Images b, d, f, h, j, l, n & p are overlays in Leica software of the L4-100K or L4-33KFLAG images with GFP and DAPI images, collected sequentially to avoid cross-talk between fluors.

L4-100K can only be expressed from the L4 cassette via the P_Bi _promoter and thus its expression and regulation in Clone A1-L4 should be similar to that of GFP in Clone A1. However, L4-22K and L4-33KFLAG can also be expressed from a recently identified L4 promoter located within the L4-100K ORF (Figure 4A) and so should be constitutively expressed [40]. To confirm these patterns of expression, Clone A1-L4 cells were examined by immunofluorescence (Figure 5). As expected, L4-100K was expressed in an inducible manner (compare panels i, j with m, n). L4-33KFLAG was also detected but its expression was not repressed by Dox (compare panels k, l, with o, p) consistent with expression occurring from the L4 promoter. L4-33KFLAG fluorescence was not significantly increased in the absence of Dox compared to levels in the presence of Dox, implying either that expression of L4-33KFLAG from the inducible P_Bi _promoter is low in comparison with its expression from the L4 promoter or that expression from the P_Bi _promoter inhibits and therefore replaces expression from the L4 promoter once Dox repression is removed. In the first case, it is possible that alternative splicing of P_Bi_-derived transcripts does not favour production of 33K mRNA, although it is clear that MLTU splicing in the natural context can give 33K expression [40]. In the second case, expression from the L4 promoter may be inhibited by competition with P_Bi _for cellular transcription factors or by promoter occlusion from overriding P_Bi_-derived transcription, or else translation of 33KFLAG from L4 promoter-derived mRNAs may be inhibited in the presence of L4-100K expressed from P_Bi _because they lack a TPL sequence [21]. Although L4-22K protein expression from Clone A1-L4 was not tested as no specific antibody was available, it would be expected that this protein, which shares the same start site as L4-33KFLAG and is expressed from the same L4 promoter, should resemble L4-33K in its expression. Equivalent constructs have been shown to express functional levels of L4-22K in another context [40].

Only a low frequency of L4-100K expressing cells was detected by immunofluorescence (Figure 5m, n). This may be as a consequence of the cytotoxicity of L4-100K, although cells were fixed and stained only 3 days after Dox removal to avoid any excessive cell death due to overexpression of L4-100K. In contrast, L4-33KFLAG was detected in 100% of cells and, as expected from the FACS analysis of the uncloned A1-L4 pool (Figure 3), Clone A1-L4 did not express GFP under any conditions, unlike Clone A1 which expressed GFP in the absence but not in the presence of Dox (Figure 5c, d, g). Both these findings confirmed that the Clone A1-L4 cells were homogeneous with respect to other aspects of transgene expression. Since L4-100K expression was assessed only 3 days after Dox withdrawal, a time when the proportion of equivalently treated parent A1 cells expressing GFP ranged from 36-66% (Figure 3A & Figure 4A), the most likely interpretation of the low frequency of 100K expression is that it reflects incomplete recovery following Dox-removal and hence, under conditions where Dox has been fully removed, all cells of Clone A1-L4 should express L4-100K.

### Clone A1-L4 expresses functional levels of L4 proteins

**Figure 6:**
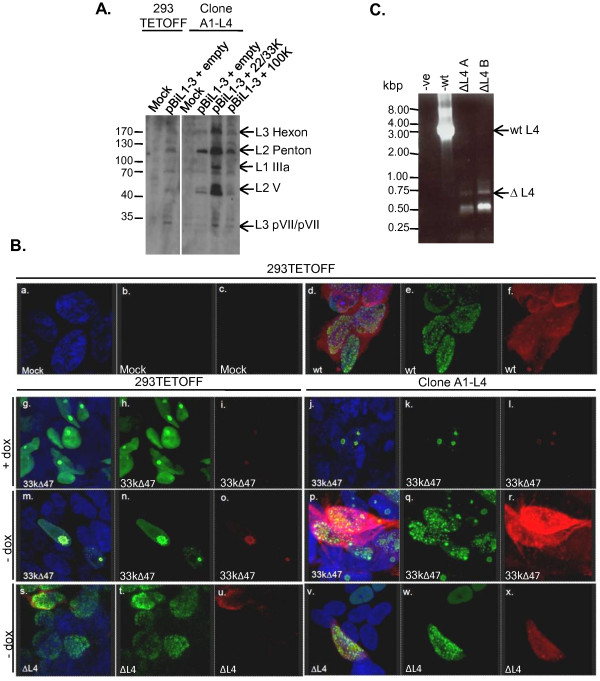
**Clone A1-L4 expresses functional levels of L4 proteins**. (A) 293TETOFF or Clone A1-L4 cells were transfected with pBiL1-3 and either pCMVFLAG (empty vector; [19]) or L4-22/33KFLAG or L4-100KFLAG expression plasmids. Cell lysates were separated on 10% polyacrylamide gels and expressed proteins detected by western blot analysis using AbJLB1 anti-late protein antisera. All lanes shown derive from the same exposure of a single blot with irrelevant lanes excised for clarity of presentation. The positions to which proteins of known molecular mass migrated are shown on the left (kDa). (B) 293TETOFF or Clone A1-L4 cells were grown in the presence (g-l) or absence of Dox (a-f & m-x) for 3 days with daily media changes prior to mock infection (a-c) or infection with P2 WT (d-f), Δ47 (g-r) or ΔL4 (s-x) viruses. Cells were fixed and stained 20 h.p.i. for Ad5 DNA binding protein (DBP) (green; b, e, h, k, n, q, t & w) or Ad5 late proteins (red; c, f, i, l, o, r, u & x) and nuclear DNA (DAPI, blue) and visualised using a Leica SP2 confocal microscope. Images a, d, g, j, m, p, s & v are overlays in Leica software of DBP, late protein and DAPI images, collected sequentially to avoid cross-talk between fluors. (C) To confirm the ΔL4 virus genotype, Ad5 23863-27086 bp region was amplified by PCR from DNA isolated from either ΔL4 or wt virus particles. ΔL4A and ΔL4B reactions contained 1 and 4 μg viral genomic DNA respectively as template; -ve, PCR negative control. The positions to which DNA size markers migrated are indicated (kbp).

Having isolated Clone A1-L4 and shown it expressed detectable amounts of L4 proteins, we now wished to show that these proteins could offer functional complementation of L4 deficiencies. We previously showed that full late gene expression from plasmid pBiL1-3, which contains the TPL and MLTU regions L1-3 under the control of the P_Bi _promoter, required L4-22K, -33K and -100K proteins *in trans *[17,19]. Therefore, to examine the functionality of the L4 proteins expressed by Clone A1-L4, the ability of this cell line to support full late gene expression from pBiL1-3 plasmid was investigated. The amount of the late proteins hexon, penton base and L2-V expressed from the plasmid was greater in Clone A1-L4 than 293TETOFF cells (Figure 6A) showing that functional levels of the L4 proteins were produced. However, this late protein expression was still limited, and could be further increased by the co-transfection of an L4-22K/33KFLAG expression plasmid to provide additional L4 protein (Figure 6A), indicating that levels of 22K and/or 33K in A1-L4 cells were limiting. Hexon was produced under these conditions, which indicated that the level of L4-100K (specifically required for hexon accumulation) was not a limiting factor. This conclusion is further supported by the fact that hexon protein levels expressed from pBiL1-3 in Clone A1-L4 cells were similar in the presence and absence of additional exogenous L4-100K (Figure 6A). Thus Clone A1-L4 cells express functional levels of L4 proteins.

To investigate whether Clone A1-L4 could complement L4 mutations in virus, linear wild-type (WT) or L4-mutant viral genomes were transfected into 293TETOFF or Clone A1-L4 cells. WT genome induced a cytopathic effect (cpe), indicative of productive infection, in both cell types. The L4-33K mutant genome Δ47 [41] induced cpe in Clone A1-L4 cells only, both in the presence and absence of Dox, but to a lesser extent under the former conditions (data not shown). This was expected as the complementing protein for Δ47, L4-33K, is produced under both these conditions (Figure 5). In contrast, cpe induced by the ΔL4 mutant genome, which requires complementation by L4-100K, -22K and -33K, was only observed in Clone A1-L4 cells in the absence of Dox (data not shown). Collectively, these data suggested that Clone A1-L4 could provide functional complementation for L4 deficiencies.

To confirm this, and to determine if infectious virus had been produced in these transfections, second passage stocks of Δ47, ΔL4 and WT viruses that had been recovered from the genome transfections described above were used to infect cells that were then analysed for early and late viral antigens by immunofluorescence. The stocks were used untitred because the complementing A1-L4 cells could not be sustained under agar as required for a plaque assay. Infections of complementing (A1-L4) and non-complementing cells (293TETOFF) were compared to confirm that the intended virus had been rescued without reversion (Figure 6B).

WT virus expressed DNA binding protein (DBP) from the E2A gene in distinctive foci within the cell nuclei that are indicative of active replication [42]; it also expressed a high level of late proteins as expected of a normally progressing infection 20 hours post-infection (h.p.i.; Figure 6B panels a-c). Δ47 virus in control 293TETOFF cells, either in the presence (Figure 6B panels g-i) or the absence of Dox (Figure 6B panels m-o), expressed large amounts of DBP but only low levels of late proteins. Moreover, DBP staining was either diffuse in the cell nuclei or present in one large replication centre per cell, indicating that infection was stalled at an early stage. Δ47 gave a similar pattern of expression in Clone A1-L4 cells under conditions of P_Bi _repression (+Dox; Figure 6B panels j-l), even though L4-33K is expressed from the L4 promoter under these conditions. This result suggests that the amount of L4-33K expressed from the L4 promoter alone is not sufficient for efficient propagation of Δ47 virus, and correlates with the lower level of cpe observed in cells transfected with Δ47 genome in the presence of Dox than in its absence. The fact that a low level of late protein expression was observed from Δ47 virus under these non-complementing conditions was expected as Δ47 genome has previously been reported to express some late proteins, in particular 100K and hexon [41]. In complete contrast, in Clone A1-L4 cells where L4 expression from P_Bi _was induced by the removal of Dox, Δ47 virus-infected cells expressed DBP that was incorporated into numerous replication centres and also produced high levels of late proteins (Figure 6B panels p-r). The number of replication centres and level of late protein staining was similar to that of WT virus, showing that the transition between early and late phase infection, for which L4-33K is required, had occurred. Taken together, these data show that Clone A1-L4 can complement an L4-33K-defective Ad5 when L4 protein expression from the cell line is induced by Dox removal.

ΔL4 virus produced numerous DBP-containing replication centres in 293TETOFF and Clone A1-L4 cells (Figure 6B panels s-u and v-x), similar to WT virus and indicating that the infection had entered the late phase. From our previous data showing that the L4 proteins are essential for late gene expression, and the need for complementation from A1-L4 cells for ΔL4 genome to cause cpe, these ΔL4 virus infections were expected to be completely defective in late gene expression in non-complementing cells. However, although most ΔL4-infected (DBP-expressing) cells showed the expected absence of late proteins in contrast to WT virus (Figure 6B panel d), a minority of the ΔL4-infected cells expressed a significant amount of late proteins. One possible explanation for this was the presence of some WT virus in the ΔL4 virus stock arising by reversion during virus isolation. To confirm that this was not the case, a PCR was performed on purified packaged DNA from a ΔL4 infection. Using primers spanning the L4 region, a band of 781 bp was amplified from ΔL4 compared to 3223 bp from WT. Absence of this 3.2 kbp band in ΔL4 reactions showed there was no detectable WT contamination in the ΔL4 stock and that ΔL4 virus defective in L4-100K, -22K and -33K had therefore been grown successfully using Clone A1-L4 cells.

## Discussion

Recent advances in our understanding of basic adenovirus biology have led to the possibility of generating vectors deleted in the regulatory proteins, L4-100K, -22K and -33K, that are now known to be required for structural protein expression. In theory such vectors would have reduced toxicity and immunogenicity as they would be unable to express the immunogenic virion proteins within transduced cells [17]. However, the propagation of L4-deficient vectors is reliant upon the development of cell lines that are able to support the growth of these replication incompetent vectors *in vitro*. Using conventional methods to generate cell lines expressing a heterologous protein results in the random integration of one or several copies of an expression cassette into the genome and the level and stability of expression is then subject to chromosomal positioning effects. In this study we deployed a LoxP/Cre RMCE strategy to overcome this problem and produce a cell line capable of inducibly expressing Ad5 L4 proteins.

The design of the RMCE system used here was carefully considered to maximise the potential for success. Firstly, humanised *Renilla reniformis *GFP was used as the reporter gene to screen LoxP parent cell lines as it has been reported that this form of GFP is less toxic than enhanced GFP or its relatives [43], thus permitting long term expression. This was an important factor as the selection process used required long periods of GFP expression without being compromised by cytotoxicity. Secondly, due to the potential toxicity of viral L4 genes to be expressed by the eventual cell line, the tetracycline-regulated promoter, P_Bi_, was chosen to drive transgene expression. This promoter, which comprises multiple copies of tetO, the recognition site for tetracycline transactivator protein tTA, and a minimal CMV promoter [44], gives expression that is tightly repressed in cell lines containg tTA in the presence of tetracycline or Dox and which can be strongly induced upon Dox withdrawal. Thirdly, the Zeocin and hygromycin resistance genes were placed under the control of the relatively weak herpes simplex virus *tk *promoter to limit potential effects on P_Bi _regulation of having a constitutive promoter located in close proximity within the inserted construct. Fourthly, the L4 cassette for transfection into GFP-expressing parent cells was designed so that the L4 mRNAs produced from it would resemble those seen in an Ad5 infection, thus maintaining the natural stability, processing and transport of these mRNAs. Finally, the absence of a promoter from the L4 cassette limited the potential for cytotoxicity due to expression of L4-100K during cell line isolation, either from unintegrated plasmid or due to random integration of the plasmid into the genome at sites other than the intended, regulated expression site.

The first stage in an RMCE protocol is equivalent to standard cell line generation methodology, and here resulted in a population of Zeocin-resistant cells with a broad range of GFP fluorescence expression characteristics from which individual cell clones were isolated and characterised. To reduce the work involved in identifying clones with appropriate expression characteristics for use as LoxP parent cell lines in this study, the Zeocin-resistant population was initially sorted by FACS, based on the level of induced GFP expression, prior to isolating individual clones. This step enriched the uncloned population for cells where the hrGFP gene had been integrated into transcripionally active regions. It would also be possible to further enhance the efficiency of the screening process by adding an additional sorting step to eliminate cells which exhibited leaky gene regulation under conditions of Dox-mediated repression.

41 LoxP parent clones were isolated and screened, 98% of which expressed GFP. The level and stringency of regulation of GFP expression was shown to be clone-specific. It has previously been reported that when this system of regulation is used in 293 cells, protein expression is detected at low levels even in the presence of Dox [34]. Here too, the P_Bi _promoter in most of the LoxP parent cell lines that exhibited what we defined as tight regulation also had a very low activity in the presence of Dox (Figure 2A). Even tighter regulation might be achieved by using as the parent cell line something other than 293 cells, since variability in basal activity of P_Bi _between cell lines has been reported [45]. However it was necessary to use 293 cells for this study as they constitutively express the Ad5 E1 proteins that are required for the propagation of E1^- ^vectors, which would form the genetic backbone of any new L4-deficient Ad5 vector construct.

Although insertion of L4 at the LoxP site of LoxP parent clone A1 should abolish GFP expression, some GFP-expressing cells were seen in the uncloned A1-L4 population. Cre-mediated recombination between LoxP sites in a circular plasmid and the target chromosome results in the inserted DNA being flanked by LoxP sites, meaning that re-excision of the inserted gene can occur by the further action of Cre recombinase [27]. This flipping effect may account for the presence of these few GFP^+ ^cells. The copy number of pCre and thus the amount of Cre recombinase in the transfected cells during isolation of an expressing cell population should reduce progressively over time through cell division, leading eventually to a stable position. Moreover, ongoing growth in the presence of hygromycin gives selection pressure for cells that do not harbour Cre recombinase as this will permit the stable retention and expression of the hygromycin resistance gene. However, there remains the possibility that in a small number of cells Cre recombinase could become stably integrated, giving rise to ongoing transgene instability. An alternative explanation for the residual GFP-expressing cells in the uncloned A1-L4 population is that non-resistant cells simply required longer exposure to hygromycin to achieve cell death. No GFP expression was observed in Clone A1-L4 cells, indicating that the recombination event that gave rise to this cell line was stable.

The level of L4-100K expression in Clone A1-L4 cells was substantial, and apparently was not limiting for complementation within the tests performed here. Previously, a cell line expressing very low levels of 100K was able to complement a 100K-mutated vector for growth to high titre [23], suggesting only low amounts of 100K are required. However, the 100K mutation employed in that study was an in-frame deletion of residues 315-543 within the 807 residue protein and so may not have been defective for all functions of this multifunctional protein. Substantial amounts of 100K are made during Ad5 infection, so higher levels of 100K are likely to be important for some aspects of its function.

Although Clone A1-L4 cells were able to provide functional levels of L4-100K, -33K and -22K in two assay systems, the amount of L4-22K and L4-33K appeared to be limiting. This was surprising as these two proteins can be expressed from a natural promoter, the L4-promoter, located within L4-100K ORF and therefore, unlike L4-100K, their expression is not subject to inhibition by Dox. However, we have previously shown that the L4-promoter is activated by early and intermediate viral proteins and by viral genome replication [40]. Since none of these was present in the functionality assay utilising pBiL1-3 in this study, activity of this promoter would have been suboptimal. The L4-promoter should have been maximally active in the genome/virus complementation experiments however, despite this, Clone A1-L4 cells did not appear to support the growth of Δ47 virus unless full expression of the L4 cassette from P_Bi _was induced by Dox removal. Whilst some cpe was observed for cells transfected with Δ47 genome in the presence of Dox, this was less than that seen when Dox was washed out of the system. This is the first time Δ47 virus [41] has been grown from its cloned genome as a complementing cell line was not previously available.

Growth of ΔL4 virus also depended on the use of complementing A1-L4 cells. When ΔL4 virus was used to infect non-complementing cells, it gave significant amounts of late proteins in a few of the cells (Figure 6B). Previously, we have shown that a genome deficient in L4-22/33K expression during the intermediate phase of infection (when these are needed to move the infectious program on into the late phase; [19]) still made some L2 penton base protein [17]. The polyclonal serum used here detects this protein, meaning that the positive late protein immunofluorescence signal in the absence of L4 complementation may be accounted for by penton base expression without full late protein expression and consequent production of virus occurring.

The LoxP/Cre RMCE strategy employed here to generate an Ad5 L4 complementing cell line could also be used to create cell lines to complement other viral vectors. Advantages of this system are that once the LoxP parent cell lines have been characterised, they represent a resource from which it is quick, easy and efficient to generate cell lines with predetermined transgene expression profiles. Isolation of useful LoxP parent lines is facilitated by prior cell sorting, which reduces the number of clones that need to be screened. As well as providing a means to isolate useful complementing cell lines, the RMCE approach should permit comparative studies of the functional properties of a series of transgenes, e.g. L4-100K mutant genes, without the confounding issue of variable expression levels.

## Conclusions

RMCE is a powerful tool in the recombineering of mammalian cell lines. This study has highlighted the potential of this approach for the generation of complementing cell lines for the growth of gene-deleted virus vectors by utilizing one of a series of LoxP parent 293 cell lines to generate an Ad5 L4-complementing cell line that was able to support the propagation of L4-mutant Ad5 viruses. The generation of further Ad5 complementing cell lines from any of the LoxP parent cell lines isolated here should be facilitated as the cell lines obtained will have predetermined expression properties.

## Methods

### Construction of pLoxPhrGFPzeo (pLoxPGFP) and pShuttle-100/22/33KFLAG

These plasmids have been briefly reported as peripheral to a previous study [40] but are described here in detail. pLoxPhrGFPzeo (pLoxPGFP) was generated by modification of pBiEGFPPacI [19]. The eGFP gene was removed by SpeI/HindIII digest and a LoxP adaptor molecule with NheI (5') and XbaI (3') compatible ends (CTAGCGGGGGCGCCGGGATCGATATATAACTTCGTATAGCATACATTATACGAAGTTATTTAATTAAGGGAAGCTTGGGT) inserted together with the NheI/HindIII fragment from phrGFP-N1 (Clontech) to generate pLoxPhrGFP. Orientation of the LoxP site was checked by sequencing. The Zeocin resistance gene (Zeo^R^) and SV40 polyadenylation sequence was amplified from pZeoSV2+ (Invitrogen) and digested with MluI/NheI while the thymidine kinase promoter (P_TK_) was amplified from pTKHyg^R ^(Clontech) and digested with MluI. The two digested PCR products were then cloned into pLoxPhrGFP to generate pLoxPhrGFPzeo, with hrGFP under control of the P_Bi _promoter and Zeo^R ^under P_tk _control.

The LoxP-L4 shuttle plasmid (pShuttle100/22/33KFLAG) was constructed from pBiEGFPPacI [19]. The P_TK_-Hyg^R ^cassette from pTKHyg^R ^(Clontech) was amplified and inserted into pBiEGFPPacI which had been digested with SapI and then treated with DNApolI (Klenow fragment) to blunt the termini. The resulting plasmid, pBiEGFP-HYG^R ^was then further modified by exchanging the P_Bi_-EGFP expression cassette for the LoxP adaptor molecule above to create a promoterless hygromycin-selectable plasmid, pLoxPshuttle. The L4 cassette for insertion into this plasmid comprised the Ad5 tripartite leader (TPL; Ad 6049-6089, 7111-7182, 9644-9733) and 109 bp intron sequence downstream of leader 3 (Ad 9734-9842), joined to Ad5 L4 sequence from 198 bp upstream of 100K ORF (Ad 23863-27086) (Figure 4B). The TPL (Ad 6049-9731) was amplified by RT-PCR from mRNA isolated from cells infected with Ad5 wt300 virus using a 5' primer containing a PacI recognition site. The 5' end of the intron was generated by amplification of Ad5 9275-9868 from Ad5 wt300 genomic DNA using a 3' primer with SalI and HindIII recognition sites. The XhoI site at position Ad 9699 present in both PCR products was used to join them. This was then ligated into pLoxPshuttle as a PacI/HindIII fragment to generate pShuttle-TPL. The 3' end of the intron and part of the L4-ORF (Ad 23863-26504) was amplified from pE2BS, a clone of Ad5 wild-type BamHI (21562) - SpeI (27082) fragment, using a 5' primer with a SalI recognition site. The 3' end of the L4 cassette, including the sequence for a C-terminal FLAG tag, was excised from pCMV33KFLAG [17] as a HindIII/XbaI fragment. The HindIII site at position Ad26328 was used to join the two fragments prior to inserting into pShuttle-TPL at PacI/XbaI to give pShuttle100/22/33KFLAG, containing a promoterless L4 cassette (Figure 4B).

### Generation of LoxP parent and A1-L4 cell lines

293TETOFF cells (Clontech) were maintained in DMEM supplemented with 10% (v/v) Tet-system approved fetal calf serum (FCS; Clontech) and 100 μg/ml geneticin (G418; Melford). To obtain LoxP parent lines, cells were seeded into poly-D-Lysine coated 6-well plates at a density of 1.5 × 10^6 ^cells/well prior to being transfected with 1 μg pLoxPGFP using Lipofectamine2000 (LF2000) at a ratio of 1 μg DNA: 3 μl LF2000. 24 h post transfection, cells were analysed under UV light for the expression of GFP before the media was changed for media supplemented with 60 μg/ml Zeocin (Invitrogen) and 100 ng/ml doxycycline (Dox; Sigma). Cells were selected for Zeocin resistance for 28 days. Cells were then grown in the absence of Dox for 7 days, with daily media changes, to induce GFP expression and then trypsinised and resuspended in serum-free DMEM at a concentration of 5 × 10^5 ^cells/ml for sorting into high and low GFP populations using a FACSVantage SE (Becton Dickinson) with 488 nm argon excitation laser and 530 nm narrow band pass filter in the normal-R sort mode. FACS data was analysed using WinMDI version 2.9. Sorted cells were seeded into 6 well plates at low density in the presence of Zeocin and Dox and individual LoxP parent cell clones isolated.

To obtain A1-L4 cells, LoxP parent Clone A1 was transfected with 1 μg pShuttle100/22/33KFLAG together with 500 ng Cre recombinase expression plasmid (pCre; [pBS185, Invitrogen]) using LF2000. From 24 h post-transfection, cells were maintained in media supplemented with 100 μg/ml hygromycin (Roche) and 100 ng/ml Dox to isolate hygromycin-resistant clones. The location of the inserted L4-cassette with respect to the P_Bi _promoter was confirmed by PCR from genomic DNA using primers specific for the promoter and for TPL leader 3.

### Southern blot analysis

Genomic DNA was isolated from cells using Tri-reagent (Sigma) according to the manufacturer's instructions. 20 μg DNA was digested with EcoRI or KpnI, resolved through 1% (w/v) agarose gel and transferred to Hybond-N nylon membrane (GE Healthcare) alongside similarly digested pLoxPGFP (1 ng, mixed with 19 μg salmon sperm DNA) as a control. DNA was detected using the 763 bp HindIII fragment from pLoxPGFP, containing the GFP gene, labelled using the AlkPhos direct labelling kit (GE Healthcare).

### Generation of ΔL4 virus genome

pE2BS was modified to delete parts of the 100K, 22K and 33K ORF while retaining the E2 regulatory sequences present on the opposite strand of Ad5 genome from the L4 region. PCR with pE2BS as template was used to generate Ad 23854-24047, using a 3' primer containing HindIII/NsiI sites, and Ad 26922-27102, using a 5' primer containing HindIII/XbaI sites. These products were cloned into pE2BS as a SmaI-HindIII-SpeI fragment to generate pE2BSΔ1. The same approach was used to give Ad 24555-24853, using a 5' primer containing a PstI site, and Ad 25738-25930, using a 5' primer containing a SalI site and 3' primer containing an NheI site. These PCR products were inserted as a PstI-XhoI/SalI-NheI fragment into NsiI/XbaI-cut pE2BSΔ1 to generate pE2BSΔL4. Finally, ΔL4 virus genome was generated by ligation of the BamHI/SpeI fragment (Ad 21563-24047, 24555-24796, 25738-25930, 26922-27081) from pE2BSΔL4 to left arm (Ad 1- BamHI 21562) and right arm (Ad SpeI 27082-35938) fragments from Ad5 wt300 genomic DNA. The resulting genome thus contains substantial deletions within the L4-100K, -22K and -33K coding sequences.

### Complementation Studies

pBiL1-3NheI (pBiL1-3), pCMVFLAG, pCMV22/33KFLAG and pCMV100KFLAG, and the analysis of gene expression from pBiL1-3, have been described previously [17,19]. For virus rescue, linear genome was prepared by *Pac*I digestion of pTG3602-Ad5wt (pWT), which contains the complete wild-type Ad5 genome [46], and pTG3602-Δ47 (pΔ47), which carries the full genome with two stop codon mutations within the L4-33K ORF that result in a protein with a 47aa truncation of its C-terminus [41]. 293TETOFF cells or Clone A1-L4 cells were grown in the absence of Dox for 3 days, seeded into 6-well plates and transfected with 500 ng WT, Δ47 or ΔL4 genome using LF2000 as described above. Once extensive cpe was observed, cells and medium were harvested and subjected to three rounds of freeze thawing to generate virus stock P1, which was then passaged in fresh cells to generate virus P2. 293TETOFF or Clone A1-L4 were infected with 1/15 volume of each total P2 stock (grown in the absence of Dox) for phenotypic analysis. The mutant genotype of ΔL4 virus was confirmed by PCR analysis using purified packaged viral DNA [47] and primers hybridising to Ad genome with 5' ends at positions 23863 and 27086.

### Western blotting and immunofluorescence

Western blotting and immunofluorescence were carried out as previously described [48,49]. Proteins were detected using the following primary antibodies: anti-FLAG rabbit polyclonal serum (Sigma) at 1:1000; AbJLB1 rabbit polyclonal serum to Ad5 late proteins at 1:10,000 for western blotting and 1:1000 for immunofluorescence [19]; rabbit anti-L4-100K (W. C. Russell, University of St Andrews) at 1:10,000 for western blotting and 1:1000 for immunofluorescence; mouse anti-DNA binding protein MAb B6-8 at 1:100 [50]. Secondary antibodies used for these studies were goat-anti-mouse IgG-horseradish peroxidise (HRP) conjugate (Sigma) at 1:5000 and goat-anti-rabbit IgG-HRP (Santa Cruz) at 1:100,000 for western blotting, and Alexafluor594 goat-anti-rabbit IgG (Invitrogen) and Alexafluor488 goat anti-mouse IgG (Invitrogen) each at 1:500 for immunofluorescence.

## Competing interests

The authors declare that they have no competing interests.

## Authors' contributions

All authors conceived and designed the experiments. SJM and DCF performed the experiments. SJM and KNL analysed the data and wrote the manuscript. All authors edited, read and approved the final manuscript.

## References

[B1] Website JoGMCT http://www.wiley.com/legacy/wileychi/genmed/clinical/

[B2] Imperiale MJ Kochanek S Adenovirus vectors: Biology, design, and production Adenoviruses: Model and Vectors in Virus-Host Interactions 2004 273 335 357 Current Topics in Microbiology and Immunology 10.1007/978-3-662-05599-1_10 14674606

[B3] Yang Y Ertl HC Wilson JM MHC class I-restricted cytotoxic T lymphocytes to viral antigens destroy hepatocytes in mice infected with E1-deleted recombinant adenoviruses Immunity 1994 1 433 442 10.1016/1074-7613(94)90074-4 7533647

[B4] Yang Y Nunes FA Berencsi K Gonczol E Engelhardt JF Wilson JM Inactivation of E2a in recombinant adenoviruses improves the prospect for gene therapy in cystic fibrosis Nat Genet 1994 7 362 369 10.1038/ng0794-362 7522742

[B5] Engelhardt JF Ye XH Doranz B Wilson JM Ablation of E2a in recombinant adenoviruses improves transgene persistence and decreases inflammatory response in mouse-liver Proc Natl Acad Sci USA 1994 91 6196 6200 10.1073/pnas.91.13.6196 8016137 PMC44165

[B6] Gao GP Yang YP Wilson JM Biology of adenovirus vectors with E1 and E4 deletions for liver-directed gene therapy J Virol 1996 70 8934 8943 8971023 10.1128/jvi.70.12.8934-8943.1996 PMC190991

[B7] Hodges BL Serra D Hu H Begy CA Chamberlain JS Amalfitano A Multiply deleted [E1, polymerase-, and pTP-] adenovirus vector persists despite deletion of the preterminal protein Journal of Gene Medicine 2000 2 250 259 10.1002/1521-2254(200007/08)2:4<250::AID-JGM113>3.0.CO;2-3 10953916

[B8] Gorziglia MI Kadan MJ Yei S Lim J Lee GM Luthra R Trapnell BC Elimination of both E1 and E2a from adenovirus vectors further improves prospects for in vivo human gene therapy J Virol 1996 70 4173 4178 8648763 10.1128/jvi.70.6.4173-4178.1996 PMC190312

[B9] Hu HM Serra D Amalfitano A Persistence of an E1(-), polymerase(-) adenovirus vector despite transduction of a neoantigen into immune-competent mice Hum Gene Ther 1999 10 355 364 10.1089/10430349950018805 10048388

[B10] Amalfitano A Hauser MA Hu HM Serra D Begy CR Chamberlain JS Production and characterization of improved adenovirus vectors with the E1, E2b, and E3 genes deleted J Virol 1998 72 926 933 9444984 10.1128/jvi.72.2.926-933.1998 PMC124562

[B11] Dedieu JF Vigne E Torrent C Jullien C Mahfouz I Caillaud JM Aubailly N Orsini C Guillaume JM Opolon P Long-term gene delivery into the livers of immunocompetent mice with E1/E4-defective adenoviruses J Virol 1997 71 4626 4637 9151856 10.1128/jvi.71.6.4626-4637.1997 PMC191684

[B12] Mitani K Graham FL Caskey CT Kochanek S Rescue, propagation, and partial-purification of a helper virus-dependent adenovirus vector Proc Natl Acad Sci USA 1995 92 3854 3858 10.1073/pnas.92.9.3854 7731995 PMC42060

[B13] Fisher KJ Choi H Burda J Chen S-J Wilson JM Recombinant adenovirus deleted of all viral genes for gene therapy of cystic fibrosis Virology 1996 217 11 22 10.1006/viro.1996.0088 8599194

[B14] McConnell MJ Imperiale MJ Biology of adenovirus and its use as a vector for gene therapy Hum Gene Ther 2004 15 1022 1033 10.1089/hum.2004.15.1022 15610603

[B15] Segura MM Alba R Bosch A Chillon M Advances in helper-dependent adenoviral vector research Current Gene Therapy 2008 8 222 235 10.2174/156652308785160647 18691018

[B16] Campos SK Barry MA Current advances and future challenges in adenoviral vector biology and targeting Current Gene Therapy 2007 7 189 204 10.2174/156652307780859062 17584037 PMC2244792

[B17] Morris SJ Leppard KN Adenovirus serotype 5 L4-22K and L4-33K proteins have distinct functions in regulating late gene expression J Virol 2009 83 3049 3058 10.1128/JVI.02455-08 19176628 PMC2655540

[B18] Ostapchuk P Anderson ME Chandrasekhar S Hearing P The L4 22-kilodalton protein plays a role in packaging of the adenovirus genome J Virol 2006 80 6973 6981 10.1128/JVI.00123-06 16809303 PMC1489068

[B19] Farley DC Brown JL Leppard KN Activation of the early-late switch in adenovirus type 5 major late transcription unit expression by L4 gene products J Virol 2004 78 1782 1791 10.1128/JVI.78.4.1782-1791.2004 14747543 PMC369502

[B20] Tormanen H Backstrom E Carlsson A Akusjarvi G L4-33K, an adenovirus-encoded alternative RNA splicing factor J Biol Chem 2006 281 36510 36517 10.1074/jbc.M607601200 17028184

[B21] Hayes BW Telling GC Myat MM Williams JF Flint SJ The adenovirus L4 100-kilodalton protein is necessary for efficient translation of viral late mRNA species J Virol 1990 64 2732 2742 2335816 10.1128/jvi.64.6.2732-2742.1990 PMC249453

[B22] Cepko CL Sharp PA Assembly of adenovirus major capsid protein is mediated by a nonvirion protein Cell 1982 31 407 415 10.1016/0092-8674(82)90134-9 7159928

[B23] Hodges BL Evans HK Everett RS Ding EY Serra D Amalfitano A Adenovirus vectors with the 100K gene deleted and their potential for multiple gene therapy applications J Virol 2001 75 5913 5920 10.1128/JVI.75.13.5913-5920.2001 11390592 PMC114306

[B24] Schneider R Grosschedl R Dynamics and interplay of nuclear architecture, genome organization, and gene expression Genes & Development 2007 21 3027 3043 10.1101/gad.1604607 18056419

[B25] Ill CR Chiou HC Gene therapy progress and prospects: Recent progress in transgene and RNAi expression cassettes Gene Ther 2005 12 795 802 10.1038/sj.gt.3302524 15815698

[B26] Sullivan MJ Carpenter AJ Porter ACG A 'select and swap' strategy for the isolation of clones with tightly regulated transgenes Eur J Biochem 2001 268 1605 1612 10.1046/j.1432-1327.2001.02027.x 11248678

[B27] Van Duyne GD A structural view of Cre-loxP site-specific recombination Annu Rev Biophys Biomol Struct 2001 30 87 104 10.1146/annurev.biophys.30.1.87 11340053

[B28] Feng YQ Seibler J Alami R Eisen A Westerman KA Leboulch P Fiering S Bouhassira EE Site-specific chromosomal integration in mammalian cells: Highly efficient CRE recombinase-mediated cassette exchange J Mol Biol 1999 292 779 785 10.1006/jmbi.1999.3113 10525404

[B29] Kito M Itami S Fukano Y Yamana K Shibui T Construction of engineered CHO strains for high-level production of recombinant proteins Appl Microbiol Biotechnol 2002 60 442 448 10.1007/s00253-002-1134-1 12466885

[B30] Nehlsen K Schucht R da Gama-Norton L Kromer W BAer A Cayli A Hauser H Wirth D Recombinant protein expression by targeting pre-selected chromosomal loci BMC Biotechnology 2009 9 100 10.1186/1472-6750-9-100 20003421 PMC2804664

[B31] Bouhassira EE Westerman K Leboulch P Transcriptional behavior of LCR enhancer elements integrated at the same chromosomal locus by recombinase-mediated cassette exchange Blood 1997 90 3332 3344 9345015

[B32] Araki K Araki M Yamamura KI Targeted integration of DNA using mutant lox sites in embryonic stem cells Nucleic Acids Res 1997 25 868 872 10.1093/nar/25.4.868 9016639 PMC146486

[B33] Graham FL Smiley J Russell WC Nairn R Characterisics of a human cell line transformed by DNA from human adenovirus type 5 J Gen Virol 1977 36 59 72 10.1099/0022-1317-36-1-59 886304

[B34] Howe JR Skryabin BV Belcher SM Zerillo CA Schmauss C The responsiveness of a tetracycline-sensitive expression system differs in different cell-lines Journal of Biological Chemistry 1995 270 14168 14174 10.1074/jbc.270.23.14168 7775477

[B35] Rennel E Gerwins P How to make tetracycline-regulated transgene expression go on and off Anal Biochem 2002 309 79 84 10.1016/S0003-2697(02)00250-6 12381365

[B36] Leppard KN Mahy BWJ, van Regenmortel MHV Adenoviruses: Molecular Biology Encyclopedia of Virology 2008 1 Oxford: Elsevier 17 23 full_text

[B37] Xi QR Cuesta R Schneider RJ Tethering of eIF4G to adenoviral mRNAs by viral 100k protein drives ribosome shunting Genes & Development 2004 18 1997 2009 15314025 10.1101/gad.1212504 PMC514180

[B38] Xi QR Cuesta R Schneider RJ Regulation of translation by ribosome shunting through phosphotyrosine-dependent coupling of adenovirus protein 100k to viral mRNAs J Virol 2005 79 5676 5683 10.1128/JVI.79.9.5676-5683.2005 15827182 PMC1082770

[B39] Huang MTF Gorman CM Intervening Sequences Increase Efficiency of RNA 3' Processing and Accumulation of Cytoplasmic RNA Nucleic Acids Res 1990 18 937 947 10.1093/nar/18.4.937 1690394 PMC330348

[B40] Morris SJ Scott GE Leppard KN Adenovirus late phase infection is controlled by a novel L4 promoter J Virol 2010 84 7096 7104 10.1128/JVI.00107-10 20444889 PMC2898241

[B41] Finnen RL Biddle JF Flint J Truncation of the human adenovirus type 5 L4 33 kDa protein: evidence for an essential role of the carboxy terminus in the viral replication cycle Virology 2001 289 388 399 10.1006/viro.2001.1130 11689060

[B42] Puvion-Dutilleul F Pedron J Cajean-Feroldi C Identification of Intranuclear Structures Containing the 72k DNA-Binding Protein of Human Adenovirus Type-5 European Journal of Cell Biology 1984 34 313 322 6236979

[B43] Vaillancourt P Felts K Rogers B Chen K Ji H Sorge J The cloned, humanized green fluorescent protein from Renilla reniformis is less cytotoxic than EGFP Mol Biol Cell 2000 11 131A

[B44] Gossen M Bujard H Tight control of gene-expression in mammalian cells by tetracycline-responsive promoters Proc Natl Acad Sci USA 1992 89 5547 5551 10.1073/pnas.89.12.5547 1319065 PMC49329

[B45] Gossen M Bujard H Efficacy of tetracycline-controlled gene-expression is influenced by cell-type - commentary BioTechniques 1995 19 213 216 8527141

[B46] Chartier C Degryse E Gantzer M Dieterle A Pavirani A Mehtali M Efficient generation of recombinant adenovirus vectors by homologous recombination in Escherichia coli J Virol 1996 70 4805 4810 8676512 10.1128/jvi.70.7.4805-4810.1996 PMC190422

[B47] Hardy S Kitamura M Harris-Stansil T Dai Y Phipps ML Construction of adenovirus vectors through Cre-lox recombination J Virol 1997 71 1842 1849 9032314 10.1128/jvi.71.3.1842-1849.1997 PMC191254

[B48] Leppard KN Everett RD The adenovirus type 5 E1b 55K and E4 Orf3 proteins associate in infected cells and affect ND10 components J Gen Virol 1999 80 997 1008 10211970 10.1099/0022-1317-80-4-997

[B49] Lethbridge KJ Scott GE Leppard KN Nuclear matrix localization and SUMO-1 modification of adenovirus type 5 E1b 55K protein are controlled by E4 Orf6 protein J Gen Virol 2003 84 259 268 10.1099/vir.0.18820-0 12560556

[B50] Reich NC Sarnow P Duprey E Levine AJ Monoclonal antibodies which recognize native and denatured forms of the adenovirus DNA-binding protein Virology 1983 128 480 484 10.1016/0042-6822(83)90274-X 6310869

